# Social and Behavioral Correlates of Self-Perceived Psychological Distress in Celiac Disease During the COVID-19 Pandemic: An Exploratory Cross-Sectional Study (COVIMPACT)

**DOI:** 10.3390/nu18111731

**Published:** 2026-05-28

**Authors:** Alessandra Marenna, Francesco Monaco, Annarita Vignapiano, Francesco Valitutti, Paolo Ciambelli, Riccardo Panella, Corrado Vecchi, Luca Steardo, Giulio Corrivetti, Alessio Fasano

**Affiliations:** 1 European Biomedical Research Institute of Salerno (EBRIS), 84121 Salerno, Italy; a.marenna@ebris.eu (A.M.); annarita.vignapiano@gmail.com (A.V.); francesco.valitutti@unipg.it (F.V.); r.panella@ebris.eu (R.P.); c.vecchi@ebris.eu (C.V.); corrivetti@gmail.com (G.C.); a.fasano@mgh.harvard.edu (A.F.); 2 Department of Mental Health, Azienda Sanitaria Locale Salerno, 84121 Salerno, Italy; 3 Pediatric Unit, Department of Medicine and Surgery, University of Perugia, 06129 Perugia, Italy; 4 Department of Industrial Engineering, University of Salerno, 84084 Fisciano, Italy; pciambelli@unisa.it; 5 Narrando Srl, 84125 Salerno, Italy; 6 Center for RNA Medicine, Department of Clinical Medicine, Aalborg University, 2450 Copenhagen, Denmark; 7 Resalis Therapeutics Srl, 10121 Turin, Italy; 8 Pharmacology and Toxicology Section, Department of Neuroscience, Psychology, Drug Research and Child Health (NEUROFARBA), University of Florence, 50121 Florence, Italy; 9 Department of Health Sciences, University “Magna Graecia” of Catanzaro, 88100 Catanzaro, Italy; lucasteardo@gmail.com; 10 Mucosal Immunology and Biology Research Center, Massachusetts General Hospital, Harvard Medical School, Boston, MA 02115, USA; 11 Division of Pediatric Gastroenterology and Nutrition, Department of Pediatrics, Massachusetts General Hospital for Children, Harvard Medical School, Boston, MA 02115, USA

**Keywords:** celiac disease, gluten-free diet, COVID-19, self-perceived anxiety, social exclusion, physical activity, Firth logistic regression, public health

## Abstract

Background: Celiac disease (CeD) requires lifelong adherence to a strict gluten-free (GF) diet. During the COVID-19 pandemic, the prevailing clinical assumption was that food supply disruptions and dietary management difficulties would be the primary sources of patient distress. This exploratory cross-sectional study directly tested this assumption in an Italian CeD cohort. Methods: COVIMPACT is an exploratory observational, web-based study conducted in Italy (data collected: July–September 2024; participants retrospectively reported their experiences during the COVID-19 pandemic period 2020–2022). Participants with a confirmed CeD diagnosis were recruited through patient associations and online networks. A structured 26-item questionnaire addressed socio-demographic, nutritional, psychological, and healthcare-access domains. Descriptive statistics, chi-square bivariate analyses (Cramér’s V as effect size), and binary logistic regression were performed using R (v4.1) and Python. Results: Among 118 participants (78% female; median age 36 years; IQR 12–42), 27% reported self-perceived psychological distress. Against expectation, difficulties in accessing GF products and changes in gluten consumption showed no clear associations with distress. Instead, social exclusion showed the strongest association (Firth OR = 5.55, 95% CI: 1.80–17.09, *p* = 0.003), while reduced physical activity (Firth OR = 5.28, 95% CI: 1.86–14.99, *p* = 0.002, full model; Firth OR = 5.54, *p* = 0.001, reduced model) and negative economic impact (Firth OR = 3.77, 95% CI: 0.89–15.97, *p* = 0.071, trend) were additional associated factors. Female sex showed a non-significant trend (Firth OR = 4.21, *p* = 0.082). All estimates carry wide confidence intervals (EPV = 4.1) and should be treated as hypothesis-generating. Conclusions: These preliminary findings suggest that social exclusion and physical inactivity may be more strongly associated with self-perceived distress than dietary challenges in contexts where GF food access is structurally protected. Results are exploratory, hypothesis-generating, and should not be generalised beyond this selected Italian cohort.

## 1. Introduction

Celiac disease (CeD) is a chronic immune-mediated enteropathy triggered by dietary gluten in genetically susceptible individuals, primarily those carrying HLA-DQ2 and/or HLA-DQ8 haplotypes [1]. Exposure to gluten provokes an adaptive immune response damaging the small intestinal mucosa, resulting in villous atrophy, crypt hyperplasia, malabsorption, and a broad spectrum of systemic manifestations [2,3,4,5]. CeD is among the most prevalent lifelong autoimmune disorders worldwide, with estimates ranging from 0.7% to 2.9% of the general population. In Italy, prevalence has risen to approximately 1.6% over recent decades, driven by improved serological screening, diagnostic awareness, and national health policies [6,7].

Lifelong strict adherence to a GF diet remains the only evidence-based treatment for CeD, leading to symptom resolution and mucosal healing in the majority of patients [4,8]. However, small intestinal bacterial overgrowth (SIBO) and other microbiota-related conditions may cause persistent gastrointestinal symptoms and complicate clinical interpretation in a subset of CeD patients even under an adherent GFD [9]. Beyond mucosal recovery, dietary quality has been shown to modulate inflammatory and oxidative stress pathways across chronic immune-mediated conditions [10]. Maintaining a GFD requires consistent access to safe, affordable GF products and constant vigilance against cross-contamination. Disruptions in food availability or affordability may cause dietary lapses, persistent gastrointestinal symptoms, nutritional deficiencies, and reduced quality of life (QoL) [4,11]. Beyond nutritional challenges, CeD is associated with significant psychosocial burden, including elevated rates of anxiety and depression, partly attributable to the social and logistical constraints of dietary management [3,12].

On 11 March 2020, the World Health Organization (WHO) declared COVID-19 a global pandemic. Lockdown measures and healthcare reorganization profoundly altered food supply chains, social interactions, and access to specialist care, creating compounded challenges for individuals with chronic diseases such as CeD. Gastroenterology departments across Italy were partially redirected to COVID-19 care, significantly limiting CeD management and the follow-up of existing patients [3,13,14,15]. Studies on dietary habits during lockdown have yielded heterogeneous results: some reported worsened dietary quality and food insecurity [16,17,18,19,20,21], while others found preserved or improved adherence due to increased home cooking [22]. Prolonged confinement was also associated with increased anxiety and depressive symptoms, which represent recognized comorbidities in CeD [3,12,23].

To address these gaps, the COVID Monitoring of Patients Affected by Celiac Disease Tool (COVIMPACT) study was designed as an exploratory investigation of the behavioral and social determinants of psychological distress in an Italian CeD cohort during the pandemic. Italy represents a particularly informative case study in this context: the national healthcare system (SSN) provides individuals with CeD with monthly reimbursement for GF food products (approximately €45–€90/month depending on age and sex), a structural economic protection largely absent in most other healthcare systems. This policy may buffer Italian patients against GF food-specific financial stress during crises, enabling the detection of other determinants of distress, social, behavioral, and economic, that would otherwise be obscured by dietary hardship in less protected populations. Rather than characterizing the full epidemiology of pandemic impact across the Italian CeD population, this study offers depth over breadth: an inferential examination of which factors predicted self-perceived psychological distress in a selected cohort, with explicit acknowledgment of the exploratory nature and sample size constraints involved. The central hypothesis was that, in a healthcare context structurally protected by national GF reimbursement policy, self-perceived psychological distress would be more strongly associated with social and behavioral factors than with dietary management difficulties. Although the acute pandemic phase has passed, these findings retain clinical relevance as a basis for preparedness in future public health emergencies.

## 2. Materials and Methods

### 2.1. Study Design and Participants

Eligible participants were individuals of any age residing in Italy with a certified diagnosis of CeD (confirmed by serology and/or duodenal biopsy in accordance with standard criteria). Exclusion criteria were: (1) absence of a documented CeD diagnosis; and (2) presence of moderate-to-severe intellectual disability precluding autonomous questionnaire completion. Data collection was conducted from 5 July 2024 to 25 September 2024, following formal ethics committee approval. The questionnaire asked participants to recall their experiences retrospectively during the COVID-19 pandemic period (2020–2022). Participant identities were pseudo-anonymized using unique alphanumeric codes to ensure confidentiality and compliance with GDPR.

The initial recruitment target of 800 participants was established a priori based on a power calculation assuming a two-sided logistic regression with α = 0.05, power = 0.80, an expected event rate of 20% (anxiety/depressive symptoms), and a minimum detectable OR of 2.5 for the primary predictor. This calculation yielded an estimated requirement of approximately 200 events (EPV ≥ 10 per predictor for 8 predictors) and, assuming 25% outcome prevalence, a total sample of approximately 800 participants. The dissemination strategy was developed and piloted over an extended preparatory period prior to data collection; the questionnaire was actively administered between 5 July and 25 September 2024 through AIC official channels and social media platforms. Despite broad dissemination, the final dataset of complete and valid submissions comprised 118 participants—approximately 15% of the planned sample. This substantial shortfall reflects several compounding factors: the logistical disruption imposed by the pandemic on clinical research networks, the reorganization of healthcare services that reduced patient contact with gastroenterological units, and possible survey fatigue or digital disengagement among a portion of the CeD population under stress. As a consequence, all analyses are substantially underpowered relative to the original design and must be interpreted as exploratory and hypothesis-generating only. A post hoc sensitivity power analysis (Hsieh et al. approximation; α = 0.05, two-sided; event rate = 28.9%) indicates that, with *N* = 114 and 33 outcome events, the study has 80% power to detect odds ratios of approximately 3.05 or larger for a single binary predictor with a 50:50 split. For ORs of 2.0, power falls to approximately 42%; for ORs of 1.5, to approximately 17%. The three primary findings of the study (OR ≥ 5.28 for social exclusion and physical activity by Firth correction) fall above the 80% power threshold, suggesting they are not merely artefacts of low power; however, the economic impact predictor (OR = 3.77) and the female sex trend (OR = 4.21) are in the region of uncertain power and should be interpreted with additional caution. These figures assume a single-predictor scenario; in the full 8-predictor model, power per individual coefficient is further reduced. Regarding the choice of statistical method for the regression analyses: LASSO regularization was considered as an alternative to Firth penalized regression but was not adopted because (1) LASSO shrinkage complicates inference and confidence interval interpretation; (2) cross-validation for tuning the penalty parameter requires sample partitioning that is not feasible at *N* = 114; and (3) Firth correction is the methodological standard recommended for logistic regression with low EPV and small samples. The full model (EPV = 4.1) is retained for completeness and transparency; the reduced model (EPV = 8.2) is preferred for inference.

### 2.2. Questionnaire and Data Collection

The questionnaire comprised 26 items organized across four domains: (1) socio-demographic characteristics (age, gender, region, marital status, education, employment); (2) socio-economic impact (household economic effects, job loss); (3) nutritional and CeD-specific variables (GF food availability, pricing, dietary adherence, need to travel for GF products, changes in physical activity, GF-related painful symptoms); and (4) psychological and digital variables (self-reported anxiety and depression symptoms, social exclusion, difficulties in daily activities, device acquisition, and social media use). Physical activity was assessed using a four-level qualitative self-report item (increased ≥ 50%, decreased ≥ 50%, unchanged, or absent). This approach was chosen in preference to quantitative measures (e.g., MET-hours or minutes per week) for two reasons: (1) the retrospective, web-based administration precluded device-based or diary-based measurement; and (2) during the pandemic context, participants’ ability to estimate absolute duration or intensity was considered less reliable than directional categorical judgement. The use of a ≥ 50% threshold aimed to capture clinically meaningful changes rather than minor fluctuations. A recognised limitation of this approach is susceptibility to recall bias, particularly for the magnitude estimate implicit in the ≥ 50% criterion; future studies should employ validated instruments such as the International Physical Activity Questionnaire (IPAQ) or device-based measurement. All data were handled in accordance with current data protection regulations (GDPR). The questionnaire was developed by the research team based on a structured review of the available literature on the impact of COVID-19 on chronic disease populations [4,5,8,24] and the specific challenges of managing a GFD during periods of social restriction. Items were drafted to cover four theoretically grounded domains: socio-demographic characteristics, socio-economic impact, nutritional management, and psychosocial well-being. The instrument was reviewed for content validity by three clinicians with expertise in CeD and psychosomatic medicine prior to administration. No formal psychometric validation study was conducted; this represents a recognised limitation of the instrument (see Limitations). Regarding the psychological distress item specifically: since psychological distress was captured by a single binary item (“Did you experience symptoms of anxiety or depression during the pandemic?”). This item captures an undifferentiated self-perceived psychological burden and does not permit differentiation between anxiety and depressive syndromes, clinical severity grading, or comparison with normative data from validated instruments such as GAD-7 or PHQ-9, standard internal consistency measures such as Cronbach’s alpha are not applicable to single-item measures. Face validity of this item was established through expert review by three clinicians with expertise in CeD and psychosomatic medicine, who judged it adequate for a screening-level assessment of self-perceived distress. However, the item does not permit clinical grading, differentiation between anxiety and depression, or comparison with normative data from validated instruments such as the GAD-7 or PHQ-9. All inferences are limited to the construct of self-perceived symptomatology. The questionnaire was administered in a single wave via Google Forms; no pilot test with a subset of participants was formally documented.

### 2.3. Ethical Approval

This study involves retrospective analysis of questionnaire data collected between 5 July 2024 and 25 September 2024 under the COVIMPACT observational protocol. The study was conducted in accordance with the Declaration of Helsinki. A formal statement of acknowledgment was issued by the Ethics Committee Campania 2 on 29 May 2024 (COVIMPACT protocol version 2.1, administrative protocol number ASLSA-0120127-2024), following submission of the complete study documentation to the competent committee. Data collection commenced prospectively on 5 July 2024, after ethics approval was in place, and concluded on 25 September 2024. The questionnaire collected retrospective recall of participants’ experiences during the 2020–2022 pandemic period. The committee’s acknowledgment reflects its determination that the study complies with applicable ethical and data protection standards. Written informed consent was obtained from all participants prior to questionnaire completion; for participants under the age of 14, written consent was obtained from a parent or legal guardian, and assent was obtained from participants aged 14–17.

### 2.4. Statistical Analysis

All analyses were performed using R (v4.1) and Python version 3.10 (scipy version 1.9; statsmodels version 0.13 libraries). Continuous variables are reported as median and interquartile range (IQR); categorical variables as absolute frequencies and percentages. Bivariate associations between categorical variables were assessed using chi-square tests on contingency tables, restricted to response options with ≥5 observations per cell; Fisher’s exact test was applied for 2 × 2 tables with expected cell counts < 5. Effect size was quantified by Cramér’s V (small: V < 0.10; moderate: 0.10 ≤ V < 0.30; large: V ≥ 0.30). Statistical significance was set at *p* < 0.05.

Prior to analysis, questionnaire responses were reviewed for data quality. Duplicate and low-quality responses were identified and excluded using standard data quality criteria. Following these quality control procedures, the final analytic sample comprised 118 participants.

The primary outcome of the inferential analyses was self-perceived psychological distress, operationalized as the binary presence or absence of any self-reported anxiety or depression symptoms during the pandemic. Importantly, no standardized psychometric instruments (e.g., GAD-7, PHQ-9, HADS) were administered; this variable therefore reflects perceived symptomatology rather than a clinically validated diagnosis of an anxiety or depressive disorder, and results should be interpreted accordingly.

To identify variables independently associated with self-perceived psychological distress, an exploratory binary logistic regression model was fitted, entering the following candidate predictors simultaneously: sex (female vs. male), employment status (employed vs. unemployed), education level (university or higher vs. lower), negative economic impact (yes vs. no), GF food access difficulties (yes vs. no), reduction in physical activity ≥ 50% (yes vs. no), perceived social exclusion (yes vs. no), and CeD-related painful symptoms (yes vs. no). Odds ratios (OR) with 95% confidence intervals (CI) are reported. Model fit was assessed using McFadden’s pseudo-R^2^ and Nagelkerke’s R^2^. Complete-case (listwise) deletion was applied; participants with missing data on any model variable were excluded, retaining *N* = 114. Given the sample size and number of predictors, the model is explicitly exploratory; wide confidence intervals reflect estimation uncertainty and should not be over-interpreted. All candidate predictors were entered simultaneously in a single step based on clinical and theoretical relevance, rather than through data-driven stepwise selection, to minimize capitalization on chance. Potential collinearity between predictors was assessed descriptively using phi coefficients for binary variable pairs; the most notable collinearity was observed between female sex and social exclusion (phi = 0.245), which may have contributed to the attenuation of the sex estimate in the multivariate model. As a pre-specified robustness check to address the low EPV of the full model, a reduced logistic regression model was also fitted, retaining only the four predictors with the strongest observed associations in the full model (social exclusion, reduced physical activity, negative economic impact, and female sex). This reduced model achieves an EPV of 8.2 and serves as a verification of the directional stability of the primary associations.

## 3. Results

### 3.1. Socio-Demographic Characteristics

A total of 118 participants were included (Table 1). The majority were female (78%), with a median age of 36 years (IQR: 12–42). Most resided in Central and Southern Italy, with Lazio (33.90%), Campania (16.10%), and Puglia (12.71%) most represented. Approximately 60% were employed, and 44.91% had attained university-level education or higher. In 73.73% of participants, CeD had been diagnosed before 2020.

### 3.2. Impact on GF Diet and Food Access

Overall, 28.82% of participants reported that the pandemic negatively impacted CeD management (Table 2). Despite the structural protection afforded by the Italian SSN reimbursement policy, a non-negligible proportion of participants, approximately one in three, reported difficulties in accessing GF products locally (32.20%), and nearly two-thirds (61.01%) noted an increase in GF food costs during the pandemic period. These signals of dietary supply pressure, while present, remained limited in scope: the large majority (72.88%) maintained their GFD without increased gluten consumption, and the fraction requiring travel to a neighboring municipality to obtain GF products (24.58%) suggests that logistical barriers, though real, were not experienced as insurmountable by most participants.

### 3.3. Physical Activity and Daily Life

A reduction in physical activity ≥ 50% was reported by 27.97% of participants; a further 22.88% reported no exercise during the pandemic (Table 2). More than half perceived CeD as a barrier to normal life at least occasionally, and approximately 47% reported mild-to-severe difficulties in performing daily activities. CeD-related painful symptoms during the pandemic were reported by 18.64%, with moderate-to-severe symptoms in 9.32%.

### 3.4. Psychological Impact and Social Behavior

Symptoms of anxiety or depression were reported by 27.12% of participants (mild: 22.03%, moderate: 5.09%; no respondent reported severe symptoms) (Table 2). Perceived social exclusion was reported by 44.91%. Social media use increased in 50.00% of respondents, predominantly on Facebook (32.20%) and Instagram (13.56%). Most participants (73.73%) already owned an electronic device and did not purchase a new one.

### 3.5. Bivariate Analyses: Factors Associated with Anxiety and Depression

Bivariate analyses (Table 3) identified five significant associations with self-perceived anxiety/depressive symptoms. Social exclusion showed the largest effect (V = 0.409, *p* < 0.001), followed by reduced physical activity (V = 0.322, *p* = 0.001), female sex (V = 0.252, *p* = 0.007), and CeD painful symptoms (V = 0.233, *p* = 0.013). Female sex was additionally associated with higher rates of social exclusion (V = 0.245, *p* = 0.009), suggesting a possible indirect pathway. GF food access difficulties, education level, and increased gluten consumption showed no meaningful association (all V ≤ 0.09, *p* ≥ 0.34).

In contrast, no significant associations were observed between psychological distress and education level, GF food access difficulties, gluten consumption, or employment-related economic impact in bivariate analysis. Notably, no association was observed between increased gluten consumption and self-perceived distress in this sample (*p* = 1.000, V = 0.000); this null finding should not be interpreted as establishing the absence of such a relationship in other populations or study contexts.

### 3.6. Logistic Regression: Independent Predictors of Psychological Distress

**Figure 1:**
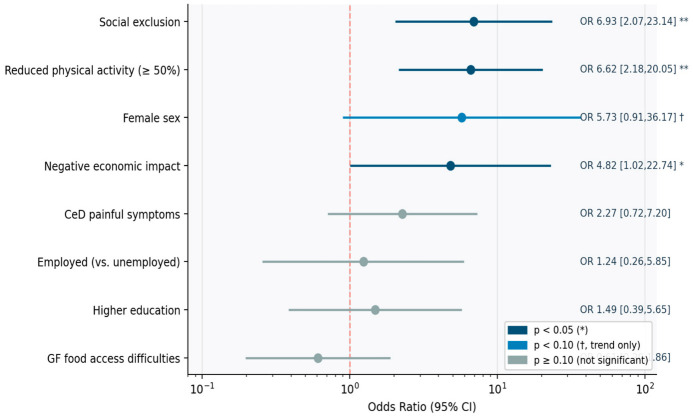
Exploratory logistic regression: associations with self-perceived psychological distress (full model, *N* = 114). Note: OR on log scale; dashed line = null (OR = 1). Dark blue: *p* < 0.05; mid-blue: *p* < 0.10 (trend only, †); grey: *p* ≥ 0.10. All estimates are exploratory; wide CIs reflect small sample size (EPV = 4.1). GF: gluten-free; CeD: celiac disease. * *p* < 0.05; ** *p* < 0.01; † *p* < 0.10 (trend only).

The exploratory logistic regression model (Table 4; *N* = 114; McFadden R^2^ = 0.34, Nagelkerke R^2^ = 0.33) showed the strongest statistical associations with self-perceived distress for social exclusion (Firth OR = 5.55, 95% CI: 1.80–17.09, *p* = 0.003, full model; OR = 6.01, *p* = 0.001, reduced model), reduced physical activity ≥ 50% (Firth OR = 5.28, 95% CI: 1.86–14.99, *p* = 0.002), and negative economic impact (Firth OR = 3.77, 95% CI: 0.89–15.97, *p* = 0.071, trend). Female sex showed a non-significant trend (Firth OR = 4.21, 95% CI: 0.83–21.25, *p* = 0.082); the particularly wide confidence interval for this estimate reflects collinearity with social exclusion (phi = 0.245 in bivariate analysis) as well as the small sample size. Employment status, education level, GF food access difficulties, and CeD-related painful symptoms did not show meaningful associations with self-perceived distress. All estimates carry substantial uncertainty and should be interpreted with corresponding caution. With 33 outcome events across 8 candidate predictors, the events-per-variable ratio (EPV = 4.1) falls below the conventional threshold of 10, indicating that parameter estimates may be unstable; this further reinforces the exploratory character of this analysis. To assess robustness, a reduced four-predictor model was tested (social exclusion, reduced physical activity, negative economic impact, female sex; EPV = 8.2). Odds ratio estimates remained highly consistent across both models (log-ratio < 0.1 for all predictors), supporting the directional stability of the primary associations despite the low EPV of the full model. Female sex, a non-significant trend in the full model (*p* = 0.082), reached significance in the reduced model (*p* = 0.058), further supporting the gender vulnerability signal discussed above. The non-significant trend for female sex (*p* = 0.082, 95% CI: 0.91–36.17) deserves measured interpretation: while this estimate falls short of conventional significance in this small exploratory sample, the direction and magnitude (Firth OR = 4.21) are consistent with the broader literature documenting higher rates of pandemic-related distress among women [25]. Our data suggest a specific vulnerability in female sex that, although at the margin of statistical significance in this exploratory sample, aligns with documented gender disparities in social isolation and psychological burden during lockdowns. Notably, in the reduced four-predictor robustness model (EPV = 8.2), female sex showed a borderline trend (Firth OR = 4.57, *p* = 0.058), providing additional support for this directional signal (Figure 1). This finding warrants targeted investigation in adequately powered future studies rather than dismissal.

As a robustness check to address the low EPV of the full model, a reduced logistic regression model was fitted retaining only the four predictors showing the strongest observed associations in the full model (social exclusion, reduced physical activity, negative economic impact, female sex; *N* = 114; EPV = 8.2). Results are summarized in Table 5. Odds ratio estimates remained highly consistent across both models: social exclusion (Firth OR = 6.01, 95% CI: 2.10–17.18, *p* = 0.001), reduced physical activity (Firth OR = 5.54, 95% CI: 1.96–15.63, *p* = 0.001), and female sex (Firth OR = 4.57, 95% CI: 0.95–21.92, *p* = 0.058). Negative economic impact showed a non-significant trend in the reduced model (Firth OR = 3.58, 95% CI: 0.85–15.09, *p* = 0.082), consistent in direction and magnitude with the full model estimate (Firth OR = 3.77, *p* = 0.071). The close correspondence of all OR estimates between the two models (log-ratio < 0.1 for each predictor) supports the directional stability of the associations, despite the exploratory character of both analyses.

## 4. Discussion

The COVIMPACT study provides exploratory evidence on the social, behavioral, and economic correlates of self-perceived psychological distress in Italian individuals with CeD during the COVID-19 pandemic. The principal finding, that dietary access difficulties were not associated with distress, whereas social exclusion, physical inactivity, and economic hardship wereis discussed below in light of methodological constraints and available evidence.

A critical interpretative caveat must be stated upfront: the composition of this sample is a fundamental moderator of the observed patterns. Participants were predominantly urban, educated, employed, patient-association members, and digitally connected, characteristics associated with substantially greater coping resources than those available to more marginalized CeD populations. It is entirely plausible that, in a sample inclusive of lower-income, rural, or elderly individuals, dietary access difficulties would emerge as a significant contributor to psychological distress. The finding that GF food difficulties are not associated with distress should therefore be read as specific to resource-rich CeD patients in the Italian healthcare context, not as a general statement about the relationship between dietary management and mental health in this condition.

A critical contextual factor distinguishes this Italian cohort from virtually all comparable international studies, and international readers, particularly those from healthcare systems without universal disease-specific dietary coverage, should note this explicitly. In Italy, all individuals with a certified CeD diagnosis are entitled by law to monthly reimbursement for GF food products through the national healthcare system (SSN), with vouchers ranging from approximately €45 to €90 per month depending on age and sex. This policy, largely absent from the US, UK, and most other healthcare systems, created a structural buffer against pandemic-related GF food cost increases for the participants in this study. The contrast with other cohorts is stark: in a Moroccan sample studied by Boutahar et al. [26], where no equivalent reimbursement exists, anxiety rates in CeD patients reached 65.3% compared to 41.6% in healthy controls. While in those contexts food costs and dietary access may have been genuine drivers of psychological stress, Italy’s welfare system appears to have neutralized this pathway, allowing the dominant behavioral and social correlates of distress to emerge: social exclusion, physical inactivity, and economic hardship operating independently of dietary management. This makes the null dietary finding not a limitation of this study but one of its most informative results, a natural experiment in what happens when food security is guaranteed and the social fabric still breaks down. A related caveat is that, without a non-celiac comparison group, it cannot be determined whether the associations observed are specific to celiac disease or would emerge similarly in the general population under equivalent pandemic conditions. Future studies should include matched non-celiac comparators to test the disease-specificity of these findings.

Monzani et al. [24] reported preserved GFD adherence in a comparable Italian lockdown cohort, consistent with our findings. The contribution of the present study is the addition of inferential analyses in an independent cohort, contextualised within Italy’s welfare system and sample characteristics. The SSN reimbursement policy may have attenuated the dietary distress pathway: while 61% of participants reported increased GF food costs, this did not translate into elevated distress, plausibly because the financial burden was partly absorbed by state subsidies. This is a methodologically informative null finding rather than a limitation.

Social exclusion emerged as the variable most strongly associated with self-perceived distress in the exploratory model (Firth OR = 5.55, 95% CI: 2.07–23.14). The significant bivariate association between female sex and social exclusion (55.8% of women vs. 25.0% of men, *p* = 0.009) suggests that gender operates partly through this pathway: the non-significant trend for female sex in the regression (Firth OR = 4.21, *p* = 0.082) likely reflects attenuation by the correlated social exclusion variable. The gender differential in social exclusion may reflect multiple overlapping mechanisms: pandemic research consistently documents that women bore a disproportionate share of domestic and caregiving burden during lockdowns, reducing opportunities for social interaction and increasing exposure to chronic low-grade stress [25]. In the CeD context, the inherently social dimensions of dietary management, sharing meals, navigating food environments, maintaining participation in social gatherings, may amplify this vulnerability. Women with CeD who already assume a greater role in domestic food preparation may have experienced heightened isolation when the normal social fabric of eating was disrupted by confinement.

Reduced physical activity showed an equally strong association with self-perceived distress (Firth OR = 5.28, 95% CI: 1.86–14.99), consistent with the extensive literature documenting the protective role of exercise in mental health and its deterioration during lockdown-related sedentariness [27,28]. More than half of participants who reported a major activity reduction also reported distress, a proportion with direct implications for clinical guidance. Negative economic impact was an additional significant predictor (Firth OR = 3.77, 95% CI: 1.02–22.74), operating through general financial stress rather than specifically through GF food affordability, as discussed above, a distinction with meaningful implications for targeted intervention.

Remote consultations are a practical component of CeD follow-up [29,30,31], but they may be insufficient as a standalone response when social exclusion is the primary correlate of distress. Evidence from eating disorders care suggests remote formats may inadequately substitute in-person support for patients with significant psychosocial burden [32,33]. Patient associations and clinical services may consider complementing telemedicine with structured social support and physical activity programs as part of emergency preparedness planning, although this recommendation is preliminary given the exploratory nature and limited statistical power of the present study.

Key limitations include: (1) small sample size (*N* = 118; EPV = 4.1 in the full model), substantially underpowered relative to the original target, producing wide confidence intervals; (2) convenience sampling through patient associations, introducing selection bias toward resource-rich, digitally connected patients. This bias is particularly relevant for interpreting the null finding on GF food access difficulties: individuals who experienced severe dietary disruption may have lacked the cognitive bandwidth, digital access, or emotional resources to complete an online questionnaire. The absence of a food access–distress association in this cohort may therefore reflect survivor selection rather than a genuine absence of such a relationship in the broader CeD population; (3) non-validated psychological outcome: the single binary item (“Did you experience symptoms of anxiety or depression during the pandemic?”) does not permit differentiation between anxiety and depressive symptoms, clinical grading of severity, or comparison with normative data. The item captures a broad, undifferentiated self-perceived psychological burden rather than a specific clinical syndrome; this limits the interpretive precision of all reported associations; (4) cross-sectional design precluding causal inference; (5) common method bias, as both distress and social exclusion were self-reported in the same questionnaire; (6) listwise deletion of missing data. The 19 incomplete cases were missing on all model variables simultaneously. Notably, some descriptive variables in Table 2 had substantially higher missingness rates: social media use was missing in 28% of responses, and the platform-specific item in 45.8%, consistent with optional items positioned at the end of the questionnaire. These variables were not included in the regression models; their high missing rates are reported for transparency and acknowledged as a limitation, indicating questionnaire non-completion rather than item-specific avoidance. Anxiety/depressive symptom rates did not differ significantly between completers and non-completers (28.9% vs. ~26%; *p* > 0.05), supporting compatibility with MCAR or MAR and the adequacy of listwise deletion in this exploratory context; multiple imputation is recommended for future confirmatory studies; and (7) absence of a healthy control group: without a non-celiac comparison cohort experiencing a similar pandemic context, it is not possible to determine whether the associations observed are specific to celiac disease or would be replicated in the general population under comparable social, economic, and behavioral constraints. The finding that social exclusion and physical inactivity predict self-perceived distress may reflect universal pandemic-era mechanisms rather than CeD-specific vulnerabilities; future studies should include non-celiac comparators to test this hypothesis. The approximately 682 individuals reached but not completing the questionnaire may represent more severely affected patients, suggesting the present cohort reflects a best-case scenario. Future studies should address these limitations with validated instruments, probability-based sampling, longitudinal designs, and healthy controls. A further generalizability constraint concerns geographic representation: over 60% of participants resided in Central and Southern Italy (Lazio 33.9%, Campania 16.1%, Puglia 12.7%), with Northern Italy substantially underrepresented. This matters because pandemic lockdown stringency, healthcare reorganization, and SSN regional distribution networks for GF products varied significantly across Italian regions. In particular, GF product reimbursement is administered at the regional level, and access may have differed between Northern and Southern regions. The null finding on GF food access difficulties may be more characteristic of well-resourced Central/Southern urban cohorts than of Northern Italian patients who, during the early pandemic, faced different supply chain disruptions. Caution is therefore warranted in extending these findings to Northern Italian contexts or to the full range of Italian regional diversity. Future studies incorporating objective nutritional, biochemical, and hematological markers would also enable more rigorous characterisation of dietary status [34]. Although robustness checks indicated directional stability of the main findings, we cannot exclude the possibility that this missingness pattern introduced additional selection or non-response bias. Furthermore, our clinical characterization relied entirely on self-reported questionnaire data; future studies should integrate objective nutritional, biochemical, hematological, immunological, or oxidative-stress markers to provide a more rigorous clinical validation [34].

## 5. Conclusions

The COVIMPACT study provides exploratory evidence that self-perceived psychological distress in Italian individuals with CeD during the COVID-19 pandemic was more strongly associated with social exclusion, physical inactivity, and economic hardship than with disease-specific dietary challenges. In a healthcare context where GF food is structurally subsidized, dietary management appears to be a relatively protected domain; it is the social and behavioral fabric of daily life that is most vulnerable to crisis-related disruption. These findings are hypothesis-generating and should not be generalized beyond the selected, resource-rich cohort studied.

Future emergency preparedness frameworks for CeD may benefit from integrating structured psychological support, physical activity promotion, and socioeconomic safety nets alongside dietary guidance, recognizing that social and behavioral wellbeing are as clinically relevant as mucosal recovery. Confirmatory studies employing validated psychometric instruments, probability-based sampling, and longitudinal designs are needed to establish causal pathways and determine the generalizability of these preliminary findings.

## Figures and Tables

**Table 1:** Socio-demographic characteristics of the study sample (*N* = 118).

Characteristic	*n* (%)
***Gender***	
Female	92 (78.0%)
Male	26 (22.0%)
***Timing of CeD diagnosis***	
Before 2020	87 (73.7%)
Between 2020 and 2022	12 (10.2%)
After 2022	8 (6.8%)
Not declared	11 (9.3%)
***Marital status***	
Married	53 (44.9%)
Single	49 (41.5%)
Divorced	2 (1.7%)
Separated	1 (0.8%)
Prefer not to declare	4 (3.4%)
Missing	9 (7.6%)
***Education level***	
University degree	30 (25.4%)
Postgraduate course	20 (16.9%)
University diploma	3 (2.5%)
High school	26 (22.0%)
Middle school	7 (5.9%)
Elementary school	9 (7.6%)
Other	15 (12.7%)
Missing	8 (6.8%)
***Occupation***	
Employed	71 (60.2%)
Unemployed	37 (31.4%)
Missing	10 (8.5%)
***Region of residence***	
Lazio	40 (33.9%)
Lombardia	20 (16.9%)
Campania	19 (16.1%)
Puglia	15 (12.7%)
Liguria	10 (8.5%)
Calabria	2 (1.7%)
Umbria	2 (1.7%)
Emilia-Romagna	1 (0.8%)
Missing	9 (7.6%)

Note: *p*-values are from chi-square goodness-of-fit tests assessing whether responses are non-uniformly distributed across the categories of each variable; they do not test associations between variables. This test is not applied to gender (binary, reference category only) or region of residence (descriptive only). These within-variable tests have limited interpretive value in a descriptive context and are reported for completeness. CeD: celiac disease.

**Table 2:** Questionnaire responses by domain (*N* = 118).

Item/Response	*n* (%)
***Socio-economic status***	
Pandemic impact on household economy (positive)	21 (17.8%)
Pandemic impact on household economy (slightly negative)	59 (50.0%)
Pandemic impact on household economy (negative)	26 (22.0%)
Pandemic impact on household economy (strongly negative)	2 (1.7%)
Missing	10 (8.5%)
Pandemic caused job loss (yes)	6 (5.1%)
Pandemic caused job loss (no)	102 (86.4%)
Missing	10 (8.5%)
***Celiac disease management***	
Pandemic negatively impacted CeD management (yes, considerably)	9 (7.6%)
Pandemic negatively impacted CeD management (yes, to some extent)	25 (21.2%)
Pandemic negatively impacted CeD management (no)	76 (64.4%)
Missing	8 (6.8%)
GF food price increase (yes, to some extent)	54 (45.8%)
GF food price increase (yes, considerably)	18 (15.2%)
GF food price increase (no)	36 (30.5%)
Missing	10 (8.5%)
Difficulty finding GF food (yes, considerably)	7 (5.9%)
Difficulty finding GF food (yes, to some extent)	3 (2.5%)
Difficulty finding GF food (yes, slightly)	28 (23.7%)
Difficulty finding GF food (no)	70 (59.3%)
Missing	10 (8.5%)
Lockdown increased gluten consumption (yes, considerably)	7 (5.9%)
Lockdown increased gluten consumption (yes, to some extent)	1 (0.8%)
Lockdown increased gluten consumption (yes, slightly)	13 (11.0%)
Lockdown increased gluten consumption (no)	86 (72.9%)
Missing	11 (9.3%)
Traveled to another municipality for GF food (yes)	29 (24.6%)
Traveled to another municipality for GF food (no)	78 (66.1%)
Missing	11 (9.3%)
***Health status***	
Pre-existing mental health diagnosis (yes, before 2020)	3 (2.5%)
Pre-existing mental health diagnosis (no)	104 (88.1%)
Missing	11 (9.3%)
Physical activity: increase ≥ 50%	9 (7.6%)
Physical activity: decrease ≥ 50%	33 (28.0%)
Physical activity: unchanged	38 (32.2%)
Physical activity: none (did not exercise)	27 (22.9%)
Missing	11 (9.3%)
CeD-related painful symptoms (none)	84 (71.2%)
CeD-related painful symptoms (mild)	11 (9.3%)
CeD-related painful symptoms (moderate)	9 (7.6%)
CeD-related painful symptoms (severe)	2 (1.7%)
Missing	12 (10.2%)
Anxiety/depression symptoms (none)	75 (63.6%)
Anxiety/depression symptoms (mild)	26 (22.0%)
Anxiety/depression symptoms (moderate)	6 (5.1%)
Anxiety/depression symptoms (severe)	0 (0.0%)
Missing	11 (9.3%)
Perceived CeD as barrier to normal life (often)	12 (10.2%)
Perceived CeD as barrier to normal life (sometimes)	29 (24.6%)
Perceived CeD as barrier to normal life (rarely)	23 (19.5%)
Perceived CeD as barrier to normal life (never)	42 (35.6%)
Missing	12 (10.2%)
Difficulties in daily activities (mild)	26 (22.0%)
Difficulties in daily activities (moderate)	27 (22.9%)
Difficulties in daily activities (severe)	3 (2.5%)
Difficulties in daily activities (none)	50 (42.4%)
Missing	12 (10.2%)
***Social isolation and digitalization***	
Felt socially excluded (yes, considerably)	19 (16.1%)
Felt socially excluded (yes, a lot)	14 (11.9%)
Felt socially excluded (yes, rarely)	20 (16.9%)
Felt socially excluded (no)	54 (45.8%)
Missing	11 (9.3%)
Purchased new electronic device (yes, PC)	12 (10.2%)
Purchased new electronic device (yes, smartphone)	1 (0.8%)
Purchased new electronic device (yes, tablet)	3 (2.5%)
Purchased new electronic device (no, already owned)	87 (73.7%)
Missing	15 (12.7%)
Increased social media use (yes, slightly; <3 h/day)	31 (26.3%)
Increased social media use (yes, considerably; >3 h/day)	28 (23.7%)
Increased social media use (no)	26 (22.0%)
Missing	33 (28.0%)
Most-used platform: Facebook	38 (32.2%)
Most-used platform: Instagram	16 (13.6%)
Most-used platform: WhatsApp	9 (7.6%)
Most-used platform: Other	1 (0.8%)
Missing (platform question)	54 (45.8%)

Note: *p*-values are from chi-square goodness-of-fit tests assessing non-uniform distribution of responses within each questionnaire item; they do not represent associations between variables. Response options with <5 observations were excluded; missing values excluded listwise. These descriptive tests have limited inferential value and are reported for transparency. GF: gluten-free; CeD: celiac disease.

**Table 3:** Bivariate associations with self-reported anxiety and depression symptoms (*N* = 114 complete cases).

Variable Pair	Anxiety+ (%)	Anxiety− (%)	Cramér’s V	*p*-Value
***Statistically significant associations (p < 0.05)***				
Female sex vs. anxiety/depression	36.0	7.1 (males)	0.252	0.007
Reduced physical activity (≥50%) vs. anxiety/depression	51.4	18.2 (others)	0.322	0.001
Social exclusion vs. anxiety/depression	49.1	10.2 (no exclusion)	0.409	<0.001
CeD painful symptoms vs. anxiety/depression	52.2	23.1 (no symptoms)	0.233	0.013
Female sex vs. social exclusion	55.8	25.0 (males)	0.245	0.009
***Non-significant associations (p ≥ 0.05)***				
Education (higher vs. lower) vs. anxiety	33.9	24.1	0.089	0.344
GF food access difficulties vs. anxiety	34.9	25.4	0.082	0.382
Increased gluten consumption vs. anxiety	30.4	28.6	0.000	1.000
Employment status vs. negative economic impact	87.9 (unemployed)	78.0 (employed)	0.089	0.342

Note: Cramér’s V: <0.10 = small, 0.10–0.30 = moderate, >0.30 = large effect size. GF: gluten-free; CeD: celiac disease.

**Table 4:** Full exploratory logistic regression: associations with self-perceived psychological distress (*N* = 114, 8 predictors, EPV = 4.1).

Predictor	OR	95% CI	*p*-Value	Sig.
Female sex	4.21	0.83–21.25	0.082	†
Employed (vs. unemployed)	1.19	0.28–5.06	0.814	
Higher education	1.42	0.41–4.96	0.585	
Negative economic impact	3.77	0.89–15.97	0.071	†
GF food access difficulties	0.65	0.22–1.90	0.436	
Reduced physical activity (≥50%)	5.28	1.86–14.99	0.002	**
Social exclusion	5.55	1.80–17.09	0.003	**
CeD painful symptoms	2.07	0.68–6.33	0.202	

Note: OR: odds ratio; CI: confidence interval. ** *p* < 0.01; † *p* < 0.10. McFadden R^2^ = 0.336; Nagelkerke R^2^ = 0.332. EPV = 4.1. All estimates exploratory. GF: gluten-free; CeD: celiac disease.

**Table 5:** Reduced logistic regression model (4 predictors; *N* = 114; EPV = 8.2): robustness check for primary associations.

Predictor	OR	95% CI	*p*-Value	Sig.
Female sex	4.57	0.95–21.92	0.058	†
Negative economic impact	3.58	0.85–15.09	0.082	†
Reduced physical activity (≥50%)	5.54	1.96–15.63	0.001	**
Social exclusion	6.01	2.10–17.18	0.001	**

Note: OR: odds ratio; CI: confidence interval. ** *p* < 0.01; † *p* < 0.10. McFadden R^2^ = 0.308; Nagelkerke R^2^ = 0.310. EPV = 8.2. Model selected for parsimony as robustness verification of Table 4 results.

## Data Availability

The anonymized dataset supporting the findings of this study is available from the corresponding author upon reasonable request, subject to approval by the Campania Sud Ethics Committee. Given the exploratory nature of the study and the importance of methodological transparency, researchers wishing to reproduce or extend these analyses are encouraged to contact the corresponding author directly. No data were withheld from analysis, and all quality control procedures applied prior to the final dataset are documented in Section 2.2.

## Abbreviations

The following abbreviations are used in this manuscript:

AIC	Associazione Italiana Celiachia
CeD	Celiac disease
CI	Confidence interval
GF	Gluten free
GFD	Gluten-free diet
HLA	Human leukocyte antigen
IQR	Interquartile range
OR	Odds ratio
QoL	Quality of life
WHO	World Health Organization

## References

[1] Lionetti E., Castellaneta S., Francavilla R., Pulvirenti A., Tonutti E., Amarri S., Barbato M., Barbera C., Barera G., Bellantoni A. (2014). Introduction of gluten, HLA status, and the risk of celiac disease in children. N. Engl. J. Med..

[2] Leo S., Leonard M.M., Valitutti F., Fasano A. (2025). Gut dysbiosis: Cause or consequence of intestinal inflammation in celiac disease?. Expert Rev. Gastroenterol. Hepatol..

[3] Cohen B.S., Lebwohl B. (2023). COVID-19 and celiac disease: A review. Ther. Adv. Gastroenterol..

[4] Bascuñán K.A., Vespa M.C., Araya M. (2017). Celiac disease: Understanding the gluten-free diet. Eur. J. Nutr..

[5] Rubin J.E., Crowe S.E. (2022). Coeliac disease. Lancet.

[6] Green P.H.R., Cellier C. (2007). Celiac disease. N. Engl. J. Med..

[7] Lionetti E., Castellaneta S., Balanzoni L., Guariso G., Nuti F., Murano A., Pontone S., Fasano A., Catassi C., SIGENP Working Group on Weaning and CD Risk (2023). Prevalence and detection rate of celiac disease in Italy: Results of a SIGENP multicenter screening in school-age children. Dig. Liver Dis..

[8] Al-Toma A., Volta U., Auricchio R., Castillejo G., Sanders D.S., Cellier C., Mulder C.J., Lundin K.E.A. (2019). European Society for the Study of Coeliac Disease (ESsCD) guideline for coeliac disease and other gluten-related disorders. United Eur. Gastroenterol. J..

[9] Sobczyk M., Porzak M., Żuraw D., Sodolska A., Oleksa P., Jasiński K. (2024). Small intestinal bacterial overgrowth—Current, novel and possible future methods of treatment and diagnosis. Prospect. Pharm. Sci..

[10] Kupczyk D., Bilski R., Szeleszczuk Ł., Mądra-Gackowska K., Studzińska R. (2025). The role of diet in modulating inflammation and oxidative stress in rheumatoid arthritis, ankylosing spondylitis, and psoriatic arthritis. Nutrients.

[11] Bascuñán K.A., Rodríguez J.M., Araya M. (2021). Pandemic effects and gluten-free diet: An adherence and mental health problem. Nutrients.

[12] Clappison E., Hadjivassiliou M., Zis P. (2020). Psychiatric manifestations of coeliac disease: A systematic review and meta-analysis. Nutrients.

[13] Catassi G.N., Vallorani M., Cerioni F., Lionetti E., Catassi C. (2020). A negative fallout of COVID-19 lockdown in Italy: Life-threatening delay in the diagnosis of celiac disease. Dig. Liver Dis..

[14] Santacroce G., Lenti M.V., Aronico N., Miceli E., Lovati E., Lucotti P.C., Coppola L., Gentile A., Latorre M.A., Di Terlizzi F. (2022). Impact of COVID-19 in immunosuppressive drug-naïve autoimmune disorders: Autoimmune gastritis, celiac disease, type 1 diabetes, and autoimmune thyroid disease. Pediatr. Allergy Immunol..

[15] Valitutti F., Troncone R., Campania Coeliac Disease Network (2021). Where have all the other coeliacs gone in 2020? Road for a 2021 catch-up with missed diagnoses. Dig. Liver Dis..

[16] Pietrobelli A., Pecoraro L., Ferruzzi A., Heo M., Faith M., Zoller T., Antoniazzi F., Piacentini G., Fearnbach S.N., Heymsfield S.B. (2020). Effects of COVID-19 lockdown on lifestyle behaviors in children with obesity living in Verona, Italy. Obesity.

[17] Werneck A.O., Silva D.R., Malta D.C., Souza-Júnior P.R.B., Azevedo L.O., Barros M.B.A., Szwarcwald C.L. (2021). Associations of sedentary behaviors and incidence of unhealthy diet during the COVID-19 quarantine in Brazil. Public Health Nutr..

[18] Marty L., de Lauzon-Guillain B., Labesse M., Nicklaus S. (2021). Food choice motives and the nutritional quality of diet during the COVID-19 lockdown in France. Appetite.

[19] Kriaucioniene V., Bagdonaviciene L., Rodríguez-Pérez C., Petkeviciene J. (2020). Associations between changes in health behaviours and body weight during the COVID-19 quarantine in Lithuania. Nutrients.

[20] Robinson E., Boyland E., Chisholm A., Harrold J., Maloney N.G., Marty L., Mead B.R., Noonan R., Hardman C.A. (2021). Obesity, eating behaviour and physical activity during COVID-19 lockdown: A study of UK adults. Appetite.

[21] Mehtab W., Singh N., Malhotra A., Makharia G.K. (2021). Impact of COVID-19 pandemic on adherence to gluten-free diet in Indian patients with celiac disease. Indian J. Gastroenterol..

[22] Flanagan E.W., Beyl R.A., Burk D.H., Spielmann G., Butler C., Martin C.K., Ravussin E. (2021). The impact of COVID-19 stay-at-home orders on health behaviors in adults. Obesity.

[23] Ludvigsson J.F., Reutfors J., Ösby U., Ekbom A., Montgomery S.M. (2007). Coeliac disease and risk of mood disorders: A general population-based cohort study. J. Affect. Disord..

[24] Monzani A., Lionetti E., Felici E., Fransos L., Azzolina D., Rabbone I., Catassi C. (2020). Adherence to the gluten-free diet during the lockdown for COVID-19 pandemic: A web-based survey of Italian subjects with celiac disease. Nutrients.

[25] Dal Santo T., Sun Y., Wu Y., He C., Wang Y., Jiang X., Li K., Bonardi O., Krishnan A., Boruff J.T. (2022). Systematic review of mental health symptom changes by sex or gender in early COVID-19 compared to pre-pandemic. Sci. Rep..

[26] Boutahar K., Ihbour S., Hadi K., Kaoutar K., Chetoui A., El Kardoudi A., Najimi M., Chigr F. (2022). Anxiety and associated factors during the COVID-19 pandemic confinement in the Moroccan adult celiac disease population. Port. J. Public Health.

[27] Wolf S., Seiffer B., Zeibig J.-M., Welkerling J., Brokmeier L., Atrott B., Ehring T., Schuch F.B. (2021). Is physical activity associated with less depression and anxiety during the COVID-19 pandemic? A rapid systematic review. Sports Med..

[28] Carroll N., Sadowski A., Laila A., Hruska V., Nixon M., Ma D.W.L., Haines J. (2020). The impact of COVID-19 on health behavior, stress, financial and food security among middle- to high-income Canadian families with young children. Nutrients.

[29] Siniscalchi M., Zingone F., Savarino E.V., D’Odorico A., Ciacci C. (2020). COVID-19 pandemic perception in adults with celiac disease: An impulse to implement the use of telemedicine. Dig. Liver Dis..

[30] Elli L., Leffler D., Cellier C., Lebwohl B., Ciacci C., Schumann M., Lundin K.E.A., Chetcuti Zammit S., Sidhu R., Roncoroni L. (2024). Guidelines for best practices in monitoring established coeliac disease in adult patients. Nat. Rev. Gastroenterol. Hepatol..

[31] Haimi M., Lerner A. (2024). Utilizing telemedicine applications in celiac disease and other gluten-free-diet-dependent conditions: Insights from the COVID-19 pandemic. Healthcare.

[32] Murphy-Morgan C., Brown R., Love C., Branley-Bell D. (2024). “Some distance between us”: A UK mixed methods study exploring experiences of remote care for eating disorders during COVID-19. Front. Psychiatry.

[33] Termorshuizen J.D., Watson H.J., Thornton L.M., Borg S., Flatt R.E., MacDermod C.M., Harper L.E., van Furth E.F., Peat C.M., Bulik C.M. (2020). Early impact of COVID-19 on individuals with self-reported eating disorders: A survey of ~1,000 individuals in the United States and the Netherlands. Int. J. Eat. Disord..

[34] Mądra-Gackowska K., Szewczyk-Golec K., Gackowski M., Hołyńska-Iwan I., Parzych D., Czuczejko J., Graczyk M., Husejko J., Jabłoński T., Kędziora-Kornatowska K. (2025). Selected biochemical, hematological, and immunological blood parameters for the identification of malnutrition in Polish senile inpatients: A cross-sectional study. J. Clin. Med..

